# Accidents, diseases and health complaints among seafarers on German-flagged container ships

**DOI:** 10.1186/s12889-023-15943-x

**Published:** 2023-05-26

**Authors:** Nora Annelies Bilir, Lorenz Scheit, Martin Dirksen-Fischer, Claudia Terschüren, Robert Herold, Volker Harth, Marcus Oldenburg

**Affiliations:** 1 Institute for Occupational and Maritime Medicine (ZfAM), University Medical Center Hamburg-Eppendorf, Seewartenstr. 10, 20459 Hamburg, Germany; 2 Clinic for Internal Medicine, Bundeswehrkrankenhaus Hamburg, Lesser Str. 180, 22049 Hamburg, Germany; 3 Hamburg Port Health Center, Beltgens Garten 2, 20537 Hamburg, Germany; 4 Faculty Life Sciences, Hamburg University of Applied Sciences (HAW Hamburg), Ulmenliet 20, 21033 Hamburg, Germany

**Keywords:** Accidents, Diseases, Health complaints, Seafarers, Container ships

## Abstract

**Background:**

For seafarers on the high seas health hazards are various and due to the setting also specific. The spectrum of job-related health impairments and accidents is mainly influenced by the maritime characteristics. The aim of this study is to assess the kind of accidents and the frequency of diseases and health complaints among seafarers on German container ships by evaluating medical log books.

**Methods:**

A systematic analysis of 14,628 medical entries from 95 medical log books of 58 container ships under German flag from 1995 to 2015 was performed. This monocentric retrospective and descriptive study used information on accidents, diseases and health complaints among different occupational groups and medical treatment procedures for the analysis and evaluation.

**Results:**

The analysis showed that more than one third of all consultations with the Health Officer on board are related to internal (33.7%) and surgical (31.3%) symptoms. Almost twenty percent of consultations were due to respiratory infections (19.6%) and accidents (17.9%). Accidents represented the most frequent reason for unfitness for sea service (31.2%). Based on occupational categories, most injuries occurred among deck crew (22.5%), followed by ratings working in the engine room (18.9%). In 106 cases, telemedical contact with a physician ashore was necessary. In total, 15 seafarers had to be evacuated from the ship for further medical treatment onshore. Medicine/drug application was the most common therapeutic measure on board, accounting for 77% of all consultations.

**Conclusions:**

The high proportion of health complaints and accidents among seafarers shows that there is a need to optimize medical care at sea and accident prevention, e.g. by standardized treatment algorithms or improving the medical training of Health Officers. The development and introduction of a digital patient file to record medical treatments on the vessels could also improve medical documentation on board.

## Background

The working conditions on a ship are subject to special hazards, which in their entirety are not comparable with other occupational activities on land, e.g. the risk of falling due to the deck surface moving in all three dimensions in rough sea. Today, there are still specific health risks associated with living and working on board, some of which have remained unchanged over the centuries (e.g. kinetosis due to ship movements) and some of which are due to more modern conditions (e.g. air-conditioning systems on board) [1]. Current health hazards particularly arise from an increased risk of accidents on damp and slippery shipboard surfaces, ladders, stairs and gaps with a risk of falling, not only during swells and storms [2]. In the long run, shift work on board also means irregular night work—across time zones and above all at night—and thus working against the circadian rhythm. This can lead to a variety of health complains such as "sleep disturbances, gastrointestinal problems, mental disorders (depression), cardiovascular diseases, obesity, and others" [3].

High responsibility, social deprivation, and "being on your own far away from family and friends" on the high seas increase stress and are typical health risks for seafarers. Due to the cramped conditions on board, there are few opportunities for sports and leisure activities to maintain wellbeing and good health. In the event of illness, medical care on board is primarily limited to the medicines and medical supplies available on board and the medical skills of the Health Officer. Based on the Maritime Labor Convention of the International Labor Organization (ILO), it is mandatory to "provide seafarers, as far as practicable, with health protection and medical care generally available to workers ashore" (Title 4: Standard A4.1).

On container ships under German flag, a ship's doctor is only required for more than 100 people on board and a voyage of more than three days (§6 paragraph 1 SchBesV 2013). Consequently, a nautical officer, the so-called Health Officer, is in charge to provide medical care for the whole crew on board. The latter obtains his medical expertise through an initial four-week training course and medical refresher courses in 5-years intervals in which all relevant medical skills must be learned and refreshed [4]. For medical treatment on the high seas, a standardized on-board pharmacy and a medical treatment room are mandatory. In the event of a medical consultation being required on board, a radio telemedical service is worldwide available for 24/7. In case of medical urgency, an evacuation from the ship can also be a treatment option.

The Health Officer must record all medical treatment measures provided to seafarers on board in a medical log book. Personal details, symptoms and the applied treatment are documented. This documentation of medical issues can be used to assess the frequency of accidents, diseases and health complaints as well as shipboard medical treatment. Up to date, there is no comprehensive epidemiologic study on seafarers' health impairments on German container vessels. A study by Faesecke et al. (2010) evaluated 23 medical log books and 3,124 entries of shipboard treatments in the period from 1988 to 1993. In this survey an anatomical distribution of the diseases was observed with a focus on the head and neck with almost 50% of the documented cases. The most frequently affected organ systems were the skin (25.0%), followed by the musculoskeletal (19.0%), gastrointestinal (10.5%) and the respiratory system (7.5%) [5].

The present study aims to estimate the type and frequency of accidents, diseases and health complaints as well as the applied medical treatment by evaluating medical log books on German-flagged container ships from 1995 to 2015. This study focused on German container ships, as in 2017, out of a total of 334 German-flagged merchant ships, 113 were container ships, representing a majority of 33.8% of the total German fleet. Considering the proportion of container ships in gross tonnage with 8,432,304 gross tonnages (GT—87.6% of the total gross tonnage) within the German merchant fleet, container ships have a high relevance, both in the German and in the international maritime industry.

The importance of this study for healthcare practice lies in the fact that by determining the frequencies of various diseases, diagnostic and treatment approaches could be optimized, for example through better training of Health Officers. In addition, this study can serve as an important source for the adjustment of national and international legal requirements for minimum standards in training and medical equipment on board. In particular, the medical equipment of the ship's pharmacy can be tailor made adapted based on the present study results.

## Methods

This study is based on the analysis of ships’ medical log books in the period from September 1957 to December 2015, which are archived at the Institute of Occupational and Maritime Medicine (ZfAM) in Hamburg, Germany. In addition, crew lists of the container ships were partially available which contained important supplementary information such as personnel data and nationality of the crews. The complete dataset includes 708 medical log books from 343 ships with a total of 136,926 book entries.

To define the dataset used for the study, the factors of nationality (German flag), ship type (container vessel), period of medical record entries (1995—2015), and medicine chest (German standard) were selected as inclusion criteria. The contents of the ships’ medicine chest are generally binding on all vessels flying the German flag and are then considered a German certified on-board pharmacy. The equipment varies depending on the shipping area and number of people on board. Incomplete data in the medical records were also defined as an exclusion criterion in this study.

**Fig. 1:**
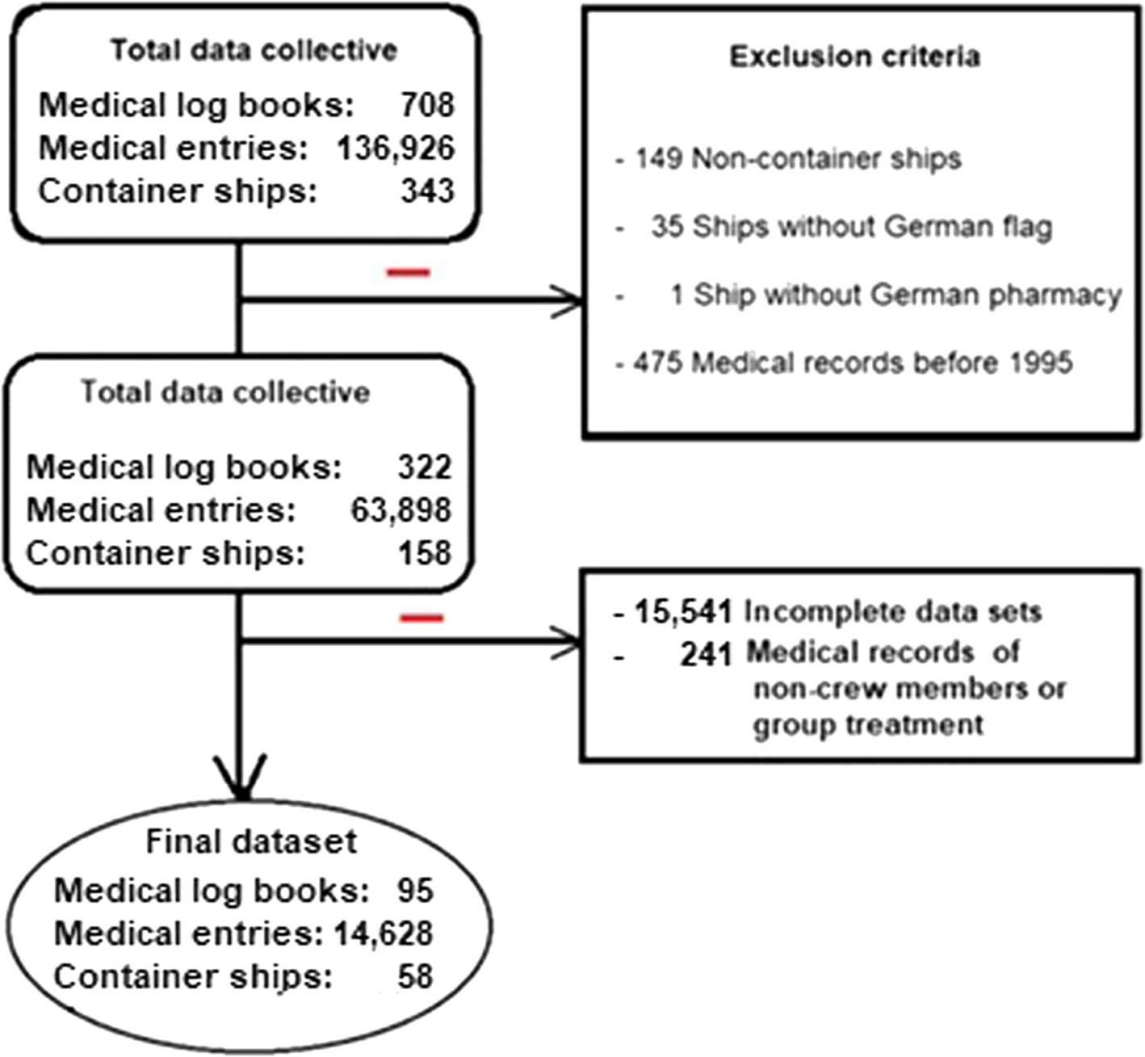
Flow chart for selection of included medical entries

Based on the above-mentioned selection criteria, out of the 343 ships, 301 ships sailed under the German flag, of which 158 ships belonged to the container ship type. In the defined period of these 158 container ships, 322 medical log books with 63,898 entries were found. Furthermore, 144 entries related to passengers and 97 entries to group treatments were excluded from further analysis. Adding the further inclusion criterion (uniform German on-board pharmacy and complete patient data) the final data set encompassed 95 medical log books with a total of 14,628 medical record entries from 58 container ships (Fig. 1).

The personal data of the available medical entries were pseudonymized by a six-digit identification code. This was necessary to assign health complaints to single seafarers and to avoid double-counting due to repetitive consultations in respect of the same health disorder. The log book entries were linked with the respective ship and entered into an Access database together with crew lists and accident reports. The health status documented by the Health Officer in each case was assigned to the seafarers’ identification code using the World Health Organization's (WHO) International Statistical Classification of Diseases and Related Health Problems (ICD-10).

Additional demographic personnel information (name, date of birth, sex, occupational group onboard), number of consultations and type of therapy were entered into the database. A statistically descriptive evaluation was performed, as well as subgroup differentiation of symptoms according to ranks. For the statistical evaluation and weighting of the relative frequency distribution of complaints, a so-called standard ship's crew for German container ships was taken as the basic constant. The standard crew defined in this way consists of 25 seafarers per container ship, of which 4 are nautical officers (NO 16%), 6 deck ratings (DR 24%), 3 technical officers (TO 12%), 4 engine ratings (ER 16%), 3 crew members (DM 12%), 2 galley personnel (GP 8%), 2 trainees (T 8%) and 1 other (4%). The evaluation of consultation frequencies was related to the proportion of different ranks according to the standard crew. The difference between the observed and the proportion-adjusted consultation frequency was calculated.

In addition, information on contacting telemedical advice ashore and being unfit for sea service was recorded. The ethics committee of the General Medical Council for the city of Hamburg (Ethikkommmission der Ärztekammer Hamburg) approved the present study (registration number WF-078/13).

For the input of the data a specified data entry mask was used, which was developed in Microsoft Access 2010. Another database was insert in Microsoft Excel 2010. For the statistical analysis SAS® (SAS Institute, Cary, NC, US) was applied. A descriptive statistical analysis of the ascertained data was performed.

## Results

### Demographic data of the total collective

The included 14,628 consultations were attributable to a total of 4,678 crew members. 158 were female (3.4%) and no gender was defined in nine crew members (0.2%).

**Fig. 2:**
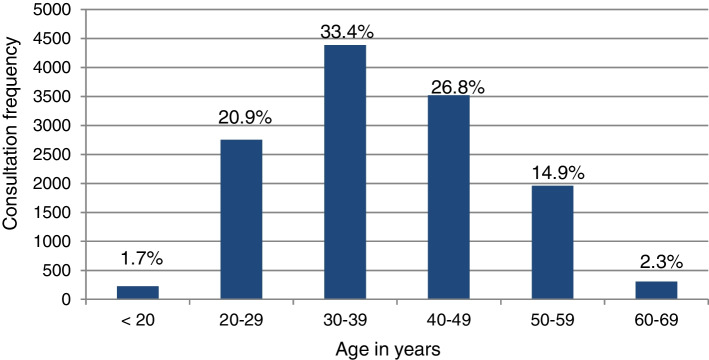
Consultation frequencies according to age

Birth records were used to calculate the age at the time of consultation. At the time of the consultation, the average age of the seafarers was 38.3 years (standard deviation (SD) 10.7 years). The frequency of consultation was highest in the group of 30–39 year old seafarers, followed by the group of 40–49 year old seafarers, seafarers between 20–29 years, 50–59 years, 60 – 69 years and below 20 years (Fig. 2).

The evaluation of nationality was possible in 3,085 male crew members. The largest proportion of seafarers came from Europe (1,408 (45.6%)), followed by Asia (1,389 (45.0%)) and Oceania (288 (9.3%)). The high proportion of Kiribati seafarers from Oceania in the present sample was due to the recruitment practices of individual German ship-owners who hired seafarers from these countries as a matter of priority (Table 1).

**Table 1:** Study population with demographic and occupational data in relation to consultation frequency

		**N**	**%**
Consultations		14,628	-
Crew members		4,678	-
Sex (*N* = 4,678)	Male	4,511	96.4
	Female	158	3.4
	Undefined	9	0.2
Nationality (*N* = 3,089)	**European**		
	- German	964	31.2
	- Polish	367	11.9
	- Croatian	11	0.4
	- Other European countries	66	2.1
	**Asean**		
	- Filipino	1,380	44.7
	- Indonesian	6	0.2
	- Syrian	2	0.1
	- Indian	1	< 0.1
	**Oceanic**		
	- Kiribati	278	9.0
	- Tuvaluan	9	0.3
	- Fijian	1	< 0.1
Ranks ^a^ (*N* = 4,511)	Nautical Officers (NO)	776	17.1
	Deck Ratings (DR)	1,032	22.7
	Technical Officers (TO)	564	12.4
	Engine Ratings (ER)	686	15.1
	Crew members with rotating jobs on deck and engine (DM)	479	10.6
	Galley Personnel (GP)	496	10.9
	Trainees (T)	411	9.0
	Other	92	2.0

^a^ Rank distribution of male seamen

The distribution of ranks among male seafarers revealed, in descending order, deck ratings, nautical officers, and engine ratings as the three most represented groups in the present collective. Technical officers (TO) corresponded to 12.4% of the seafarers. The crew members with rotating jobs, the galley/ service personnel and cadets/trainees were equally represented (Table 1).

A further analysis of the occupational subgroups showed that the group with the highest frequency of consultations (14.5%) is that of Able Bodied Seamen (AB), who belong to the crew ratings and are predominantly from the Asian region.

### Reasons and frequencies for medical consultations

Based on the assigned person ID, it was possible to identify seafarers’ consultations due to follow-up consultations because of the same health disorder. This shows that 38.5% of the medical entries corresponded to a first contact. Second contacts were registered in 21.7% of the cases. In one exceptional case, 27 consultations were documented for a seafarer.

**Fig. 3:**
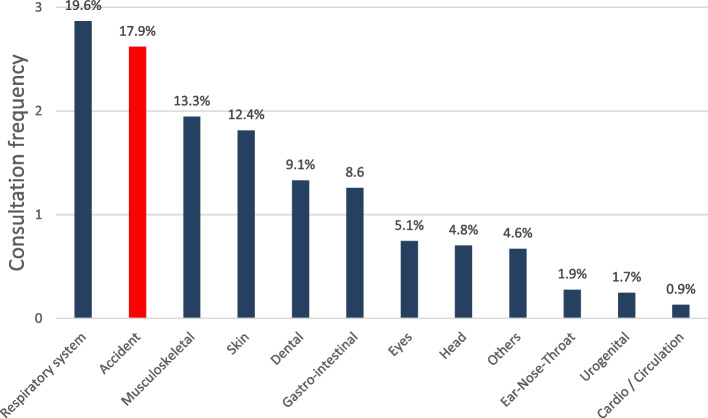
Consultation frequencies according to organ- and accident-related symptoms

Concerning all documented consultations, symptoms of the respiratory system (19.6%) were the most common reason for seeking the advice of Health Officers, followed by accidents (17.9%), and musculoskeletal impairments (13.3%). Skin symptoms (12.4%) and dental complaints (9.1%) also represent frequent reasons for medical consultation in fourth and fifth position. The distribution of the other organ systems in descending order was as: dermatological, dental, ophthalmic, urogenital and ear-nose-throat (Fig. 3).

If the organ-related reasons for consultation were assigned to the medical disciplines, a focus on internal medicine (33.7%) and surgery (31.3%) became apparent.

The four most frequent reasons of respiratory complaints to consult the Health Officers were analyzed in more detail. Among the 2,742 documented entries of respiratory complaints, the most common reason was a common cold (38%), followed by sore throat (20%), cough (17%), and flu or influenza (15%). The other complaints described as specific were pharyngitis (3%), tonsillitis (2%), and sinusitis (1%).

Among the gastrointestinal complaints, heartburn (23.3%) and diarrhea (21%) were most common.

As the second most frequent medical reason for consultation, it was not possible to divide the musculo-skeletal complaints into individual disease patterns, since the lay-diagnoses of the Health Officers were too imprecise in this context. For this reason, the musculo-skeletal complaints were considered as a unity. Nevertheless, a qualitative analysis showed that the diagnosis of back pain was most frequent, but without recording an exact assignment of the back region.

In 1,736 medical records, complaints of the skin resulted in the consultation of the Health Officer. With 28% of the cases, skin rash was the most frequent reason for consultation followed by not specified (18%), skin infection (15%), mycosis (13%), abscess (13%), pruritus (7%), sunburn (3%), dry skin (2%) and Herpes simplex (1%). Skin problems were most common among the deck ratings (23.1%), followed by nautical officers (17.1%), engine ratings (15.8%), and galley personnel (15.8%).

In 1,331 (9.1%) of all consultations, oral and dental complaints represented the fourth most frequent group of medical entries. Further differentiation of complaints was not possible based on the medical entries. Table 2 illustrates the reasons for consultation in relation to different ranks and the difference between the observed and the proportion-adjusted consultation frequencies. Particular high prevalence war found for respiratory complaints in nautical officers, muskuloskelettal complaints in deck ratings and skin complaints in galley personnel.

**Table 2:** Reasons for medical consultations by ranks

		**NO**^**a**^	**DR**	**TO**	**ER**	**DM**	**GP**	**T**	**Other**
Respiratory complaints	observed^b^	20.0	25.2	14.9	11.4	10.0	11.1	5.5	1.9
	difference^c^	4.0	1.2	2.9	-4.6	-2.0	3.1	-2.5	-2.1
Muskuloskelettal complaints	observed	14.5	28.4	12.9	14.8	11.2	11.8	4.8	1.7
	difference	-1.5	4.4	0.9	-1.2	-0.8	3.8	-3.2	-2.3
Skin complaints	observed	17.1	23.1	9.5	15.8	10.7	15.8	6.0	2.0
	difference	1.1	-0.9	-2.5	-0.2	-1.3	7.8	-2.0	-2.0
Complaints of the mouth and periodontium	observed	18.0	27.2	11.2	14.1	10.0	14.1	3.9	1.2
	difference	2.0	3.2	-0.8	-1.9	-2.0	6.1	-4.1	-2.8
Accidents	observed	8.3	22.5	12.8	18.9	14.3	9.9	10.6	2.7
	difference	-7.7	-1.5	0.8	2.9	2.3	1.9	2.6	-1.3

^a^ *NO* Nautical Officers, *DR* Deck Ratings, *TO* Technical Officers, *ER* Engine Ratings, *DM* Crew members with rotating jobs on deck and engine, *GP* Galley Personnel, *T* Trainees

^b^ observed: Relative frequency of complaints in the different ranks [%]

^c^ difference: Difference between observed and expected frequency according to the proportion of ranks in a standard ship's crew of German container vessels [%]

### Accidents and their consultation frequencies

**Fig. 4:**
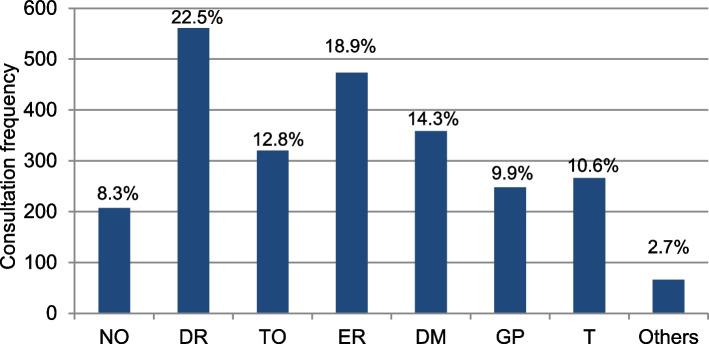
Consultation frequencies by rank due to accident-related complaints. NO = Nautical Officers, DR = Deck Ratings, TO = Technical Officers, ER = Engine Ratings, DM = Crew members with rotating jobs on deck and engine, GP = Galley Personnel, T = Trainees

Shipboard accidents accounted for 2,499 of the medical consultations. In 22.5% of those accidents deck ratings and in 18.9% engine room ratings were involved, followed by crew members with rotating jobs (14.3%), technical officers (12.8%), trainees (10.6%), galley personnel (9.9%), nautical officers (8.3%), and others (2.7%) (Fig. 4).

**Fig. 5:**
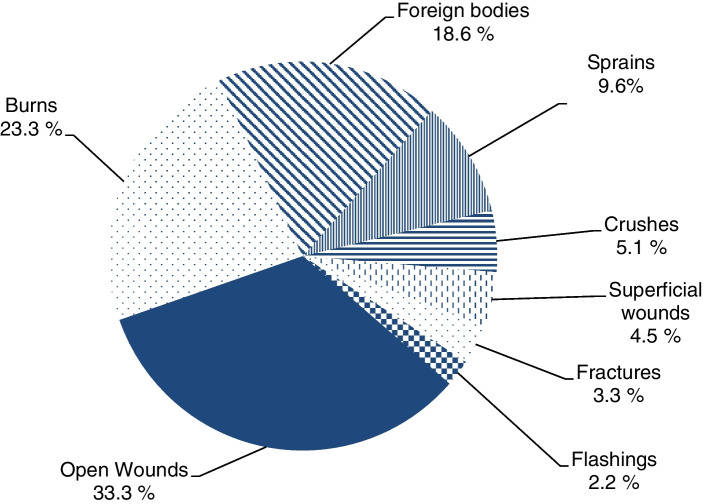
Types of accidents

Of the recorded accidents, 1,817 (72.7%) could be assigned to an affected body region. The assignment was anatomically divided into head/neck, upper body, arms, hands, legs and feet. It can be seen that the head/neck region (36.3%) and the hands (44.0%) account for the majority of all injuries. The other body regions affected were arms (10.6%), legs (6.3%), upper body (2.4%), and feet (0.4%). The 1,817 accident reports listed above were analyzed in respect of the type of injury. The analysis shows that the three most often injuries were open wounds (e.g., cuts) burns and foreign body accidents (Fig. 5).

106 documented medical complaints led to a contact to a telemedical doctor, representing less than 1% of all consultations. In 21.7% of these cases, accidents were the reason for the telemedical consultation. Diseases of the skin (16.0%) and genitourinary region (11.3%) were the second and third most frequent reason for seeking telemedical advice, respectively. The other consultation reasons are listed in descending frequency: health complaints of the gastrointestinal tract (9.4%), musculo-skeletal (8.5%), others (7.5%), cardiovascular (6.6%), respiratory symptoms (5.6%), complaints of the oral-dental region (3.7%), allergies (1.8%), eye complaints (1,8%), pains (1.8%), complaints of the head (0.9%) and of the ear, nose and throat area (0.9%).

In 46.2% of the medical entries, no information had been registered about the fitness for sea service of the health impaired person. In the remaining 53.8% of entries, it was found that in 6,520 (45.9%) entries a fitness for duty was documented and in just under 900 (7.9%) unfitness for sea service was recorded. The largest proportion of unfitness for sea service was related to shipboard accidents (31.2%), followed by musculo-skeletal (15.8%), respiratory symptoms (12.9%), complaints of the gastro-intestinal tract (11.3%), skin (7.2%), others (7.1%), heart/circulation (3%), urogenital (3%), eye (2.4%) and head complaints (2.1%) and ear-nose-throat symptoms (0.5%).

A further analysis showed that a total of 15 sick or injured seafarers had to be evacuated from the ship, for example, by a rescue helicopter. Of these 15 seafarers, 5 were seriously injured and the other 10 showed various causes like complaints of the head region, the urogenital region, the skin, the gastro-intestinal tract, cardiovascular problems, allergic reaction, others, one unknown reason and dead.

The evaluation of medical treatment shows that 18,723 therapeutic interventions were implemented in the documented consultations. A treatment can also include several therapeutic measures. The eight most common therapies were the administration of medication (77.1%), followed in descending frequency by dressing application (6.8%), wound care (5.1%), support/elastic bandaging (1.1%), cooling (1.1%), foreign body removal (1.0%), irrigation/gurgle (0.4%) and heat treatment (0.4%).

## Discussion

The analysis of the medical log books in the present study showed that the most common health complaints of seafarers on German-flagged container ships were respiratory symptoms with a proportion of 19.6%. In descending order, accident-related complaints occurred (17.9%), followed by musculo-skeletal (13.3%), dermatological (13.3%), oral-dental and gastrointestinal symptoms. The frequency distribution of complaints on board from this study is similar to two other studies conducted in 2009 and 2016. Schlaich et al. (2009) examined the frequency of infectious diseases on board German-flagged container ships using 49 medical log books from 2000–2008 and observed that respiratory infections accounted for the majority of illnesses [1]. D´Esposito (2016) examined 7,700 cases of ill seafarers from a Hamburg maritime practice from 1998 to 2011 and showed that the main diagnoses were, in descending order, accidental injuries, gastrointestinal diseases, back problems, and cardiovascular diseases [6].

A comparison of our results with the results of the study by Faesecke et al. (1993) shows different frequencies of complaints documented in the medical log books on board. The difference concerning the frequency of skin complaints (25.0% vs 13.3% in the present study) can be explained on the one hand by the fact that at the time of data collection 30 years ago there was often no intensive sun protection of employees, e.g. in respect to UV radiation. The currently lower frequency of skin complaints among shipboard crews was surprising as during the past decades an increasing proportion of Asian seafarers on board has been employed who, according to Fitz-Patrick, have a less light-sensitive skin color. The more frequent occurrence of respiratory problems today compared to the 1990s can probably be explained by the rapid succession of ports today with rapid climate zone changes and problems with air conditioning.

Generally, a comparison of the relative frequencies of various health impairments among seafarers determined in this study with available disease prevalence from land-based high-risk occupations (such as construction workers, carpenters or cooks) is not possible. Further, there are different methods of data collection and evaluation (medical lay examination without professional confirmation, documentation of symptoms and no diagnosis). Comparing the frequency distribution of complaints of the male general population on the basis of ICD-10 diagnoses from statistics of general practitioner outpatient treatment from 2015, the study findings are consistent with the frequency observed ashore in respect of respiratory diseases (19.9%). Musculo-skeletal complaints are more often in men ashore (23.1%). One possible reason could be the effect due to ship movements at sea in sense of a training of proprioceptors. Chronic diseases, such as high blood pressure or metabolic diseases, which also have a high relevance in the general population ashore, apparently played only a minor role on board or were at least not recognized as a medical problem by the Health Officer. Furthermore, this difference may be caused by a preselection due to the required medical fitness test, in which relevant chronic diseases can lead to an exclusion for service on ships.

Concerning all complaints of seafarers, internal diseases represented the most frequent reason for health consultation (33.7%). Respiratory complaints documented in the medical entries are frequently based on symptom descriptions, so that an etiology or precise diagnosis often cannot be assigned. Generally, causes of acute respiratory illness are often viral, fungal, or bacterial infections, irritant gases or dusts [7]. Possible influencing factors that lead to frequent respiratory complaints on board container ships could also be dry air caused by air conditioning systems and germ distribution through air circulation, as well as the favored dust exposure due to the technical-specific environment of ships. Von Hahn, for example, describes a longer survivability of influenza viruses at lower humidity as often found aboard [8]. Another contributing factor to respiratory irritation is the different microclimates on board, e.g. temperature and humidity in the engine room differ from other ship compartments and from the geographic outdoor climate on deck. In addition to the handling of paints and varnishes that irritate the respiratory tract, exposure to pollutants from exhaust gases, especially sulfur oxides from heavy fuel oil, must also be taken into account [9]. In our study population, nautical officers showed a + 4% higher relative consultation frequency for respiratory symptoms than expected, whereas engine ratings demonstrated a -4.6% lower consultation frequency. This could possibly be related to the fact that nautical officers have to switch between indoor and outdoor activities more frequently due to their work activities, whereas engine ratings work predominantly sheltered from the weather on the ship.

In addition to respiratory diseases, gastrointestinal complaints were common, with heartburn occurring in 23.3% and diarrhea in 21.0%. Different studies describe that the on-board lifestyle is associated with unhealthy behaviors such as malnutrition, smoking, alcohol consumption, stress, lack of exercise, and high-risk behaviors, which may promote the occurrence of gastrointestinal diseases [10]. A study on the level of knowledge and understanding of food hygiene among on-board personnel showed that cooks, kitchen assistants and service personnel in particular had the lowest level of knowledge, although it is precisely these personnel who is responsible for preparing meals. Whether this circumstance is responsible for the relatively high number of diarrheas among seafarers was not investigated in this study [11].

Cardiovascular-related complaints were relevant in only 0.8% of all consultations in the study population. This low proportion is surprising, since several studies have shown that cardiovascular diseases are the most common cause of death among seafarers on board ships [12] and a large proportion of medical emergencies are also due to cardiovascular events [13]. A possible explanation for this low proportion could be that cardiovascular diseases are often chronic and asymptomatic and the symptoms of acute events, such as headache in hypertension or complaints of angina, are misinterpreted as heartburn. Consequently, cardiovascular-related complaints were probably not recognized by the treating Health Officer.

Musculo-skeletal complaints represented the third most frequent reason for consultations (13.3%). Based on the analysis of the medical log books, it is evident that back pain accounted for the main reason for consultations among musculo-skeletal complaints. The recording of consultation frequencies of various complaints by medical laypersons in our study does not allow a comparison with available prevalence studies. Nevertheless, a comparison of the specific diagnoses between seafarers and the general population working on land can be drawn in available morbidity studies. A study by Hansen et al. (2004) examined the morbidity of seafarers and fishermen in relation to different ranks using standardized hospitalization rates (SHR) [14]. The central and national registry of hospitalized patients in Denmark served as a reference. Here, seafarers had a higher hospitalization rate for trauma and poisoning, and a higher mortality among ratings than officers.

A former study by Oldenburg et al. (2015) estimated discharge diagnoses due to non-cancerous diseases among German seafarers using SHR [10]. This study showed a decreased SHR of 0.91 (95%CI 0.88–0.93) for musculoskeletal disorders among seafarers, but also for respiratory, cardiovascular, uro-genital, and gastrointestinal disorders. Furthermore, a subgroup analysis of ranks revealed significantly increased SHR for all diseases among galley personnel, suggesting an occupationally increased health risk of this working group.

Since no further specification, for example, in cervical, thoracic or lumbar spine, emerged from the documentation, no more precise classification can be made. The symptom of "back pain" is equally found in all rank groups. Musculoskeletal disorders with back pain arise multifactorially from genetic predisposition, lifestyle, social environmental factors, individual training, performance levels, stress perception and resistance [15]. Bridge personnel such as captains and nautical officers have a more static, sedentary job with potential lack of movement and poor posture, which can lead to back pain. In contrast, engine room and deck ratings are more likely to have a high level of physical strain with incorrect loads, dislocations, and poor posture as the cause for back pain. However, the reasons for the musculo-skeletal complaints of the seafarers observed can only be assumed due to a lack of documentation and differentiation of the complaint patterns.

Skin complaints were even a common reason for consultations the Health Officer (13%). The complaints were mostly skin rashes, itching, mycoses, skin infections or sunburn. Deck ratings (23.1%) and galley personnel (15.8%) were the occupational groups on board that most frequently suffered from skin complaints. The frequency of skin complaints among deck ratings can possibly be explained by their contact with skin-irritating substances such as varnishes, paints and solvents. In the case of galley personnel, this is reinforced by the fact that they frequently work with water and moisture.

Comparing seafarers' skin-related reasons for seeking medical advice with the prevalence of skin diseases would not be valid. A comparison of the present frequency of skin-related reasons for counseling with the available prevalence of skin complaints in different occupational risk groups on land is also not possible. Based on a recent cross-sectional study, a significantly increased prevalence (OR 1.67) of actinic keratosis as a putative UV-induced sequela was found in seafarers compared to a general land-based population [16–18].

In the present study, accident-related complaints are the second most frequent reason for health consultation, accounting for 17.9%. Generally, seafaring is regarded as high-risk occupation for accidents due to slippery surfaces and demanding working and living conditions on board over 24 h per day [19]. An additional study revealed that the standardized mortality rate (SMR) in seafarers was 1.3 times higher than in other land-based employees (men SMR 132 (95% CI 118–147), women SMR 125 (95%CI 99–157)) [19].

A differentiated examination of the total of 1,823 accidents shows that, in descending order, open wounds, burns and foreign body injuries make up the majority of accidents. These findings confirm the study results by Brauer (2009), which showed that the most common cause of accidents were traumata, cuts, and burns in an analysis of 7,200 documented medical treatments of seafarers between 1995 and 2007 [20]. The specific accident hazards to which seafarers are exposed on board exist in many ways, for example, on the ground of the floors, stairs, ladders, doors and gaps [2], which can be very dangerous, especially during storms and rain due to the wetness. Because of these sources of danger, "slipping, tripping and falling" often occur on board [21]. Other causes of shipboard accidents can be falls into the cargo hold or during heavy seas and storms [22, 23]. Corresponding to the different hazards at specific workplaces, for example, burns or cuts show up more frequently among galley personnel, which can be attributed to the handling of knives and hot oils and liquids. In our study, galley personnel consulted the Health Officer for accidental injuries (9.9%) more frequently (+ 1.9%) than expected (8.0%). It is also evident that the preferred anatomical region of injury among galley personnel is the hands, as this is where proximity to potentially hazardous objects is greatest.

Looking at the most common injuries in the workplace "deck" shows an accumulation of sprains, open wounds and foreign body accidents, with the hands and the head/neck being the most commonly affected body regions. Among deck ratings, accidents were most frequently observed (22.5%), which corresponds to their workplace-specific accident hazards. Interestingly, when considering the proportion of the shipboard ranks the deck ratings had less often accidents (-1.5%) than expected. The highest proportion-adjusted frequency of consultations due to accidents were found among engine rates (18.9%; + 2.9% higher than expected), which generally reflects their high accident risk due to their hard physical work and dealing with many dangerous machines in engine room.

Due to fall or impact injuries, the head/neck region and hands are most exposed. Accidents caused by foreign bodies typically occur during grinding work in the area of the eyes. Among the group of engine room personnel, burns, open wounds and accidents due to foreign bodies are predominant, with the hands and head also preferred body areas. The listed accident hazards with their conceivable accident consequences reflect the great importance of appropriate work clothing and responsible work organization, because "inattention, carelessness or misjudgment of a situation on board are the greatest enemies of prevention" [24].

The occupational group cadet had 2.6% more often consultations for injury-related consultations than expected. The above-average proportion of accident-related consultations in the occupational group cadet (10.6%) could be due to the younger age of the seafarers with little work experience, which leads to misjudgment of the dangers in the workplace.

With less than 1%, the documented telemedical consultants represent a comparatively small proportion. The most important reasons were accidents and diseases of the skin or urogenital region. These data indicate possible medical uncertainties of the Health Officers particularly in the treatment of heavy skin or urogenital diseases.

Furthermore, in this study a relatively low rate of unfitness for sea service (7.9%) was found. Since in 46.2% of the medical entries no respective documentation was carried out, the real proportion cannot be defined with certainty, which shows the high relevance of consistent and complete written documentation. The proportion of persons defined as unfit for duty was most frequently due to accidents, followed by musculoskeletal and respiratory complaints, which is to be expected given the above-described demanding working conditions on board container ship.

The limitations of this study are described below. The description of symptoms/complaints documented in the medical log books are entered by the Health Officer. Since the symptom description was not carried out by a physician, but by a medical layperson without well-founded differential diagnostic knowledge, there is a risk of an inaccurate or shallow diagnosis and must be critically questioned. Furthermore, it is also not possible to recognize from the documentation whether a physical examination has taken place.

When reviewing the medical log books, it became apparent that important data were often not recorded. A lack of documentation in the medical records by the Health Officer therefore represents another limitation of this study. The numerous incomplete data that were excluded from further analyses refer to the entries made by different Health Officers on different container vessels; a targeted distortion of data was not recognizable. However, it cannot be ruled out that some rare diseases were not included in the present analysis, but given the large number of entries included, a significant bias through the exclusion of incomplete data is not likely.

Some health complaints and illnesses were treated by the seafarers themselves using over-the-counter medicines or from their own medicine cabinets. It can be assumed that seafarers treat certain symptoms such as respiratory complaints, headaches, and musculoskeletal complaints themselves on board using medicines sold over-the-counter and therefore there is no entry in the medical record. This phenomenon would lead to an underestimation of the frequency of complaints requiring treatment on board.

Due to the low proportion of women of 3.4% in the present study collective, this study mainly focused on male seafarers on container ships under the German flag and therefore does not convey sufficiently valid statements about the health complaints of female seafarers. Due to the existing data structure of the present dataset, no differentiated evaluations of the disease prevalence according to demographic factors were possible. This was not the focus of this study. In general, subsequent maritime studies are recommended that aim to show demographics associated with different diseases.

There are some important implications of the present results for health services on board. The requirements for the medical equipment in the ship's medicine chest are discussed as an ongoing process. In Germany, the medical chest is also kept up to the standard of the latest medical progress. The standard of medical equipment is established by the Committee for medical equipment in the maritime shipping sector ("Ausschuss für medizinische Ausstattung in der Seeschifffahrt"). The committee mainly consists of competent experts for maritime medicine in Germany as well as of deck officers and can use the present data from medical log books of container vessels. Consequently, tailor-made adjustments can be made to the medical equipment – ​​nationally and internationally (e.g. the International Medical Guide for Ships from the International Maritime Organization (IMO)).

Due to the high frequency of respiratory diseases, accidents and musculoskeletal disorders, it is important to better focus on these most common complaints. The preventive medicine during the medical education of ongoing ship officers plays an important role to reduce the risk for illness and injury among seafarers. More targeted treatment of seafarers should be envisioned by adapting medical training for medical officers. Medical documentation should be optimized and digital medical documentation protocols should be considered. The present results from this unique occupational group provide insight into the specific health risks on board. In further studies, for example, the influence of Covid 19- or other infections on seafarers could play a role, as well as the focus on different demographic subgroup analyses.

## Conclusions

Based on the evaluation and data interpretation of the study, possible improvement options in medical management on board German-flagged container ships can be derived. The main aspects of possible improvements are the medical training of the Health Officers, the digitalization of the medical log books, and the implementation of standardized algorithms in the performance of certain medical measures.

The time interval between the required medical refresher courses is 5 years. This is a relatively long period during which medical knowledge can be lost. Thus, shortening the time intervals between training measures could improve the level of knowledge and experience of Health Officers. Analogous to the training interval of 2 years required by the German Social Accident Insurance (Deutsche Gesetzliche Unfallversicherung) for company first aiders, a reduction of the medical training interval for nautical officers to, for example, 2 or 3 years could also lead to a qualitative improvement in medical knowledge and safety in the application of medical measures for seafarers on board.

Considering the observed frequency of complaints, a treatment focus on internal and surgical issues became apparent. In combination with the knowledge deficits in the field of internal medicine and surgery described in the study by Oldenburg et al. (2014), an adjustment of the training content could lead to an improved level of knowledge of the Health Officers [25]. The evaluation of the telemedical consultation shows that dermatological and urological complaints represent an important reason for telemedical support. The uncertainty of the Health Officers postulated here can also be taken into account by a more demand-oriented expansion of medical training.

In addition, the introduction of algorithms for certain complaints can help to ensure that diagnoses can be made or ruled out more quickly and accurately. Symptom-based algorithms could therefore be developed, which include a targeted anamnesis, a guided assessment (e.g. blood pressure measurement, temperature measurement, ECG writing, urine analysis, etc.) and recommendations for further action (e.g. telemedical consultation).

The identified problem of missing or inadequate documentation in the medical log books, which makes it difficult to evaluate the results, points to a further optimization option. Since the medical documentation available in paper form was often not or only incompletely filled out, diagnoses, findings, treatments and forms of therapy cannot always be traced. The development and introduction of an electronic digital medical record, in compliance with data protection regulations, could be a solution for this problem and is currently being discussed among maritime experts. Better documentation could also be achieved through mandatory field specifications in the medical entries. The maritime medical service of the BG Verkehr in Germany has developed a procedure for the electronic recording of health data on board. The authors are currently conducting a study to evaluate the usefulness of a digital patient record for recording medical treatments on the vessels. In total, the specific benefits of the improvements mentioned need to be evaluated in future studies as well as future studies should focus on the demographics associated with different diseases.

## Data Availability

The datasets used and/or analyzed during the current study will be available from the corresponding author on reasonable request.

## Abbreviations

ILO: International Labor Organization
SchBesV: Schiffsbesetzungsverordnung
ZfAM: Zentralinstitut für Arbeitsmedizin und Maritime Medizin
WHO: World Health Organization
ICD: International Classification of Diseases
ECG: Electrocardiogram
